# Acute tryptase determinations in NSAID-induced anaphylaxis: could we avoid drug challenges?

**DOI:** 10.1186/2045-7022-5-S3-P144

**Published:** 2015-03-30

**Authors:** Esozia Arroabarren, Antonio Rodriguez, Jose Maria Olaguibel, Blanca Garcia, Maria Teresa Aldunate, Maria Alvarez-Puebla, Sara Garrido, Susana Echechipía, Maria Teresa Lizaso, Marta Anda, Belen Gomez, Ana Tabar

**Affiliations:** 1 Complejo Hospitalario de Navarra, Pamplona, Spain; 2 Hospital Reina Sofia de Tudela, Pamplona, Spain

## Objective

To seek for possible differences in tryptase values during acute anaphylaxis induced by non-steroid anti-inflammatory drugs (NSAID) related to the suspected mechanism (selective vs non-selective reactions).

## Methods

Retrospective review of patients attending the Emergency Department (2009-2013) for a NSAID induced anaphylaxis and with a tryptase determination. Patients were split into 3 groups according to the allergy work-up Results

Selective reactions (S) were defined by positive skin results and/or negative challenges with other NSAID; non-selective (NS) reactions were defined by the existence of symptoms with several NSAID or after positive challenge results. Those cases who did not comply with the previous conditions were non-conclusive (NC). We analysed: demographics, severity of the episode (Chi Square test), and rate of tryptase higher than 11.4 mcg/l (Chi Square test), median tryptase values and timing of sample obtention (Kruskall-Wallis).

## Results

86 patients were included: S group: 36 patients, NS group: 20 patients, and NC: 30 patients. Median ages were 48, 45 and 54 years, respectively. Severity of the episodes: S group: Mild: 22.2%, moderate: 30.6% and severe: 47.2%; NS group: Mild: 30%, moderate: 35% and severe: 35%; NC group: Mild: 16.7%, moderate: 26.7% and severe anaphylaxis: 56.7% (p=0.658). The most frequent eliciting drugs were metamizole (19 cases in S group, 7 cases in NS group and 12 in NC group, respectively), acetic derivatives (7 cases in S group, 3 cases in NS group and 3 in NC group) and propionic acid derivatives (6 cases in S group, 5 cases in NS group and 12 in NC group). The median of tryptase determinations were: S group: 13.4(2.75-98.6), NS: 12.35(3.85-53.6) and NC: 13.4mcg/l (2.82-57.1) (p=0.856). Median duration of symptoms: S group: 1.5 (0.33-12), NS: 1.75 (0.5-4); NC: 1.5 hours (0.5-6h) (p=0.582).

## Conclusions

Although there have been reports of differences in tryptase releases during anaphylaxis related to different allergens, we have found no differences among the different patterns of NSAID reactions that may help us to avoid future challenges for diagnostic confirmation.

